# Low-dose computed tomography image denoising using pixel level non-local self-similarity prior with non-local means for healthcare informatics

**DOI:** 10.1038/s41598-025-10139-2

**Published:** 2025-07-11

**Authors:** Dawa Chyophel Lepcha, Bhawna Goyal, Ayush Dogra, Krunal Vaghela, Ashish Singh, K. S. Ravi Kumar, Durga Prasad Bavirisetti

**Affiliations:** 1 Department of ECE, University Centre for Research and Development, Chandigarh University, Mohali, Punjab 140413 India; 2 Faculty of Engineering, Sohar University, Sohar, Oman; 3 Chitkara University Institute of Engineering and Technology, Chitkara University, Rajpura, Punjab India; 4 Department of Computer Engineering, Faculty of Engineering and Technology, Marwadi University Research Center, Rajkot, Gujarat 360003 India; 5 NIMS School of Electrical and Electronics Engineering, NIMS University Rajasthan, Jaipur, India; 6 Department of Electronics and Communication Engineering, Raghu Engineering College, Visakhapatnam, Andhra Pradesh 531162 India; 7 Department of Computer Science, Norwegian University of Science and Technology, Trondheim, Trøndelag Norway

**Keywords:** Low-dose computed tomography (LDCT), Normal-dose computed tomography (NDCT), Image denoising, Nonlocal self-similarity (NSS), Haar transform (HT), Medical imaging, Cancer, Computational biology and bioinformatics, Diseases, Health care, Medical research, Risk factors, Engineering

## Abstract

Low-dose computed tomography (LDCT) has gained considerable attention for its ability to minimize patients’ exposure to radiation thereby reducing the associated cancer risks. However, this reduction in radiation dose often results in degraded image quality due to the presence of noise and artifacts. To address this challenge, the present study proposes an LDCT image denoising method that leverages a pixel-level nonlocal self-similarity (NSS) prior in combination with a nonlocal means algorithm. The NSS prior identifies similar pixels within non-local regions, which proves more feasible and effective than patch-based similarity in enhancing denoising performance. By utilizing this pixel-level prior, the method accurately estimates noise levels and subsequently applies a non-local Haar transform to execute the denoising process. Furthermore, the study incorporates an enhanced version of a recently proposed nonlocal means algorithm. This revised approach uses discrete neighbourhood filtering properties to enable efficient, vectorized, and parallel computation on modern shared-memory platforms thereby reducing computational complexity. Experimental evaluations on publicly available benchmark dataset NIH-AAPM-Mayo Clinic Low-Dose CT Grand Challenge demonstrate that the proposed method effectively suppresses noise and artifacts while preserving critical image details. Both visual and quantitative comparisons confirm that this approach outperforms several state-of-the-art techniques in terms of image quality and denoising efficiency.

Computed tomography (CT) has been extensively studied and utilized since its introduction in the 1970s. However, repeated CT scans are often required for image-guided interventions, CT-guided lung lesion assessments, and radiotherapy planning, all of which aim to produce high-resolution images that clearly depict soft tissues. Despite their diagnostic benefits, frequent volumetric scans significantly increase radiation exposure poses serious health risks such as radiation-induced cancers, metabolic disorders, leukaemia, and other genetic abnormalities^1^. In clinical settings, there is a strong imperative to minimize radiation exposure while maintaining sufficient diagnostic accuracy. However, reducing the radiation dose often leads to images with elevated noise levels and persistent streak artifacts which degrade image quality and compromise diagnostic reliability^2^. To overcome these limitations, numerous strategies have been explored to improve the quality of reconstructed images from low-dose CT (LDCT) including the development of optimized scanning protocols^3^, advancements in imaging hardware^4^, and the application of sophisticated image reconstruction algorithms^4–6^. Enhancing the hardware capabilities of CT systems can ultimately improve dose efficiency. Current research trends focus on dose reduction through advanced imaging techniques; particularly statistical iterative reconstruction methods combined with both projection-domain and image-domain denoising. These approaches aim to reduce computational cost, facilitate easy implementation, and maintain high image quality. Among projection-domain techniques, Filtered Back Projection (FBP) used in nearly all commercial CT scanners remains the most common. FBP operates on raw or logarithmically transformed measurements but often introduces additional noise and edge blurring^7^. Recently, significant advancements in statistical iterative reconstruction have been made by formulating objective functions that comprise two components: (a) a data fidelity term.

that models the statistics of acquired measurements, and (b) a regularization term that integrates prior knowledge. The reconstruction is then achieved by iteratively minimizing this objective function^8^. Ongoing research continues to focus on optimizing the regularization term for better results. Various image priors, such as compressed sensing^8^, dictionary learning^9^, low-rank approximations^10^, and total variation^11^, have been effectively explored. While iterative reconstruction methods show strong potential to enhance image quality, they face two major limitations: first, they are often vendor-specific, as details about scanner geometry and correction processes are not accessible to all users; second, they are prone to artifacts and require high computational resources.

Low-dose computed tomography (LDCT) imaging has gained considerable importance in the medical field, primarily due to its capability to reduce radiation exposure while still maintaining useful diagnostic quality. Despite this advantage, the significant downside of LDCT is the elevated noise and artifacts introduced due to lower radiation levels, which may obscure anatomical structures and hinder accurate diagnosis. Thus, denoising LDCT images is a vital step in medical imaging, aiming to suppress unwanted noise while preserving critical anatomical and structural information for applications in oncology, cardiology, and trauma diagnostics^12^. To address the challenges of LDCT imaging, various deep learning-based approaches have been introduced. Chen et al.^13^ proposed a deep convolutional neural network that performs patch-wise mapping from low-dose images to their normal-dose counterparts, even without access to raw projection data. Further, they introduced RED-CNN, a residual encoder-decoder convolutional neural network enhanced with autoencoder, deconvolution, and shortcut layers^14^. RED-CNN demonstrated superior performance in both simulated and clinical settings. Wu et al.^15^ developed a cascaded training network to iteratively refine denoising and mitigate artifacts caused by denoising operations using CNN cascades. Wolterink et al.^16^ employed a generative adversarial approach, training a CNN alongside an adversarial network to convert LDCT images into routine-dose images using voxel-wise loss minimization. Yang et al.^17^ took this further with a GAN incorporating the Wasserstein distance and perceptual similarity measures, yielding promising results in clinical CT imaging. Shan et al.^18^ proposed a 2D-to-3D conveying path-based convolutional encoder-decoder (CPCE) network, showcasing the benefits of spatially incorporating adjacent slice information during model training. Fan et al.^19^ introduced a quadratic autoencoder model that leverages quadratic neurons for denoising LDCT images. Their results on the Mayo dataset validated its resilience and efficiency. Zhao^20^ enhanced the traditional BM3D technique to better suit LDCT data by optimizing Wiener filter coefficients using the MMSE criterion while considering the noise spectrum. However, residual streak artifacts and edge preservation remained a challenge with this method. Trung et al.^21^ adopted a sparse representation and image decomposition strategy, while Li et al.^22^ introduced a modular parallel clone neural (PCN) network capable of accelerating the learning process without increasing model complexity. A task-oriented denoising network (TOD-Net)^23^ was proposed, employing task-specific loss functions to improve performance in regions of diagnostic interest. A probabilistic self-learning (PSL) scheme using only LDCT images to characterize both noise distribution and pixel correlation was introduced to exploit shift-invariant properties^24^. To further improve denoising performance, Trung et al.^25^ implemented deep CNNs with extended receptive fields by incorporating pre- and post-processing steps. This allowed distant pixels in the input image to enrich feature maps, thereby improving denoising results. Wang et al.^26^ introduced a convolution-free Token2Token dilated vision transformer (CTformer), which used token rearrangement and dilation to capture local and long-range dependencies while eliminating edge artifacts through overlapped inference techniques. Deep learning-based LDCT denoising networks have become widespread across multiple domains^27–30^.

Yang et al.^31^ presented a sinogram-domain LDCT denoising model utilizing local and global inner designs for loss computation. Han et al.^32^ proposed a novel GAN framework with dual encoders and a single decoder. Meanwhile, a stationary wavelet transform (SWT)-based network was introduced for frequency-specific LDCT denoising^33^, enhancing both low and high-frequency components using wavelet domain losses. To ensure optimal translation of LDCT to normal-dose CT (NDCT), Li et al.^34^ proposed an ASWCNN model. Li et al.^35^ also introduced MSFL-Net for multiscale adaptive feature extraction across varying resolutions. Wang et al.^36^ presented a dual-branch architecture combining a noise estimation network and a restoration network. U-Net-based modules were employed for aggregating multiscale noise features. Yan et al.^37^ merged convolutional dictionary learning with CNN in a framework called TLD-CDL. After pretraining on natural image datasets with dense connections and multiscale inception modules, the model was applied to LDCT images using transfer learning. Isola et al.^38^ contributed a widely-used conditional GAN (pix2pix) for image translation, which later found its way into LDCT denoising tasks. Yi et al.^39^ enhanced pix2pix by integrating a sharpness detection network. Ma et al.^40^ introduced a GAN trained with L1 loss, least squares, and structural similarity metrics. Their generator learned noise patterns in LDCT images and removed them, with L1 and SSIM helping maintain texture and sharpness. Recognizing the difficulty of acquiring aligned training pairs, a Cyclefree Cycle-GAN^41^ architecture was developed. This network, built in the wavelet residual domain, uses an invertible generator that naturally satisfies cycle-consistency constraints, eliminating the need for an additional discriminator. Du et al.^42^ emphasized two critical elements in LDCT denoising: network design and adversarial loss. They proposed DNSGAN, which separated noise suppression from structure recovery and introduced multiscale relativistic adversarial loss to preserve fine structural details. Huang et al.^43^ introduced DUGAN, a dual-domain U-Net GAN that learned differences in both image and gradient domains. This framework provided per-pixel feedback for improving global semantic accuracy while enhancing edge structures. Despite the success of many recent methods, shape artifacts still persist, especially when using multiscale techniques that struggle with information retention at coarser scales. Lepcha et al.^44^ tackled this using a constructive non-local means method combined with morphological residual processing, providing a novel solution to LDCT denoising. Han et al.^45^ developed an enhanced K-SVD approach based on image decomposition and adaptive dictionary learning, preserving structural information by processing modal components independently. Wang et al.^46^ proposed a dual-encoder architecture combining the Transformer model’s ability to grasp global context with convolution’s proficiency in capturing local patterns. This hybrid design markedly improved denoising performance. Finally, Li et al.^47^ introduced the progressive cyclical CNN (PCCNN), a simple multi-stage model that uses wavelet transformations to denoise while preserving high-frequency features. Wang et al.^48^ contributed a scale-sensitive GAN, employing an error feedback pyramid generator to heighten the denoiser’s responsiveness to artifacts and noise at varying image scales.

In this study, we propose an effective LDCT image denoising algorithm that leverages pixel-level nonlocal self-similarity (NSS) prior in conjunction with a nonlocal means approach. While conventional methods often operate at the patch level, our objective is to elevate this concept by applying NSS at the pixel level thus exploiting finer image details for enhanced denoising performance. Utilizing the pixel-level NSS prior, we introduce a method for accurate noise estimation, followed by the development of an image denoising framework based on the nonlocal Haar wavelet transform (LHWT) and nonlocal means filtering. Initially, we identify and extract uniform (i.e., structurally similar) nonlocal patches and then search for homogeneous pixels within these patches. Noise level estimation is conducted on each group of similar pixels. Local signal intensities are then estimated using the Haar transform with bi-hard thresholding, and the roughly reconstructed pixel values are restored through inverse Haar transformation. The nonlocal means algorithm proves particularly effective for images containing repetitive structured patterns. By calculating a weighted average of similar pixel values, it exploits the redundancy present in natural images to reduce noise. Unlike traditional neighbourhood filtering methods that rely on sliding window operations, our method employs a more advanced mechanism that allows for faster and more efficient implementation. To refine the denoising process, the noise-reduced pixel data is further processed by combining the nonlocal means algorithm output with global noise estimation using Wiener filtering. Subsequently, inverse LHWT is applied to generate a clean pixel matrix. These matrices are aggregated to reconstruct the final denoised image. A key innovation of our approach lies in its emphasis on pixel-level NSS, which allows for the identification of highly similar individual pixels across non-local regions. This level of granularity is more feasible and accurate than patch-based matching and enables more precise noise suppression while preserving complex textural details. By addressing pixel-level NSS directly, our method effectively captures and mitigates even subtle noise variations without compromising image quality. The complete workflow of the proposed method is illustrated in Fig. 1.

**Fig. 1:**
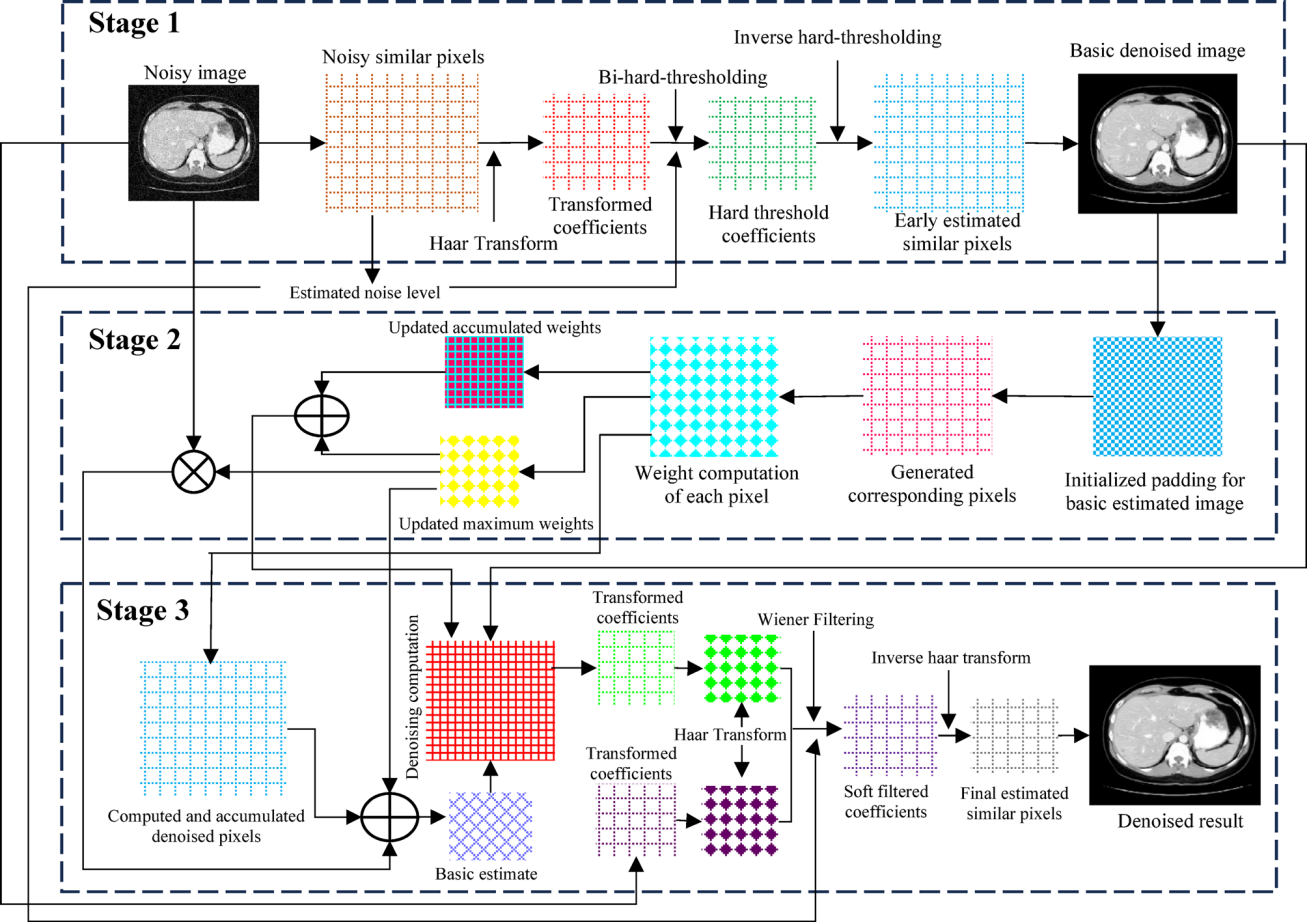
Methodological workflow of the proposed method.

The main contributions of the proposed method are summarized below.

1. This study proposes a pixel-level non-local self-similarity prior to enhance denoising in low-dose CT images. Unlike traditional approaches that focus on patch similarity, this method identifies similar pixels directly by incorporating advanced state-of-the-art techniques to enhance efficiency and significantly reduce processing time compared to existing methods.
2. This study utilizes the pixel-level non-local self-similarity (NSS) prior to accurately estimate noise levels in low-dose CT images. To enhance denoising performance, it introduces a pixel-level non-local Haar transform in conjunction with the non-local means (NLM) algorithm. The effectiveness and accuracy of the proposed method are validated using a publicly available benchmark dataset, highlighting its robustness and efficiency.
3. The proposed denoising strategy optimizes the traditional non-local means algorithm by significantly reducing its computational complexity. It achieves this through a fast, vectorized implementation designed for parallel processing, making it highly efficient on modern shared-memory computing platforms.
4. Comprehensive evaluations on benchmark datasets demonstrate that the proposed method consistently outperforms existing approaches, both visually and quantitatively, in terms of image quality and denoising accuracy.

The remainder of this study is structured as follows: “Related work” provides a brief overview of the concept of non-local self-similarity (NSS) and the non-local means algorithm. “Materials and methods” details the proposed image denoising method along with its numerical implementation. In “Experimental results and analysis”, we present extensive experimental results to evaluate the denoising performance, comparing our approach with recent state-of-the-art methods on standard benchmark datasets. Finally, “Discussion” concludes the study and outlines potential directions for future research.

## Related work

### Non-local self-similarity (NSS)

Non-local self-similarity (NSS) priors have proven highly effective in various image processing tasks such as denoising, super-resolution, texture synthesis, and inpainting. In the context of image denoising, the Non-Local Means (NLM) algorithm was one of the first methods to leverage the NSS prior^49^. NLM estimates the intensity of each pixel by computing a weighted average of all other pixels in the image, where the weights are based on the similarity between patches centered at those pixels. Although NLM operates at the pixel level, it relies on patch-based similarity, making it effectively a patch-level method. This concept was further advanced in the Block-Matching and 3D Filtering (BM3D) algorithm^50^, which applies NSS by grouping similar patches and collaboratively filtering them. BM3D assumes the grouped patches are highly similar; however, identifying perfectly matching patches across natural images is inherently challenging. To address this limitation, the current study proposes a shift from patch-level to pixel-level denoising by searching for similar pixels directly, enabling more localized and accurate noise reduction. For additional work related to this topic, readers are referred to^51^.

### Non-local means (NLM) algorithm

The Non-Local Means (NLM) algorithm represents a state-of-the-art approach for denoising, particularly effective in CT imaging under the assumption of additive Gaussian noise^52,53^. NLM^54^ performs optimally when the image contains repetitive and structured patterns. It estimates each pixel’s intensity by computing a weighted average of neighbouring pixel values, utilizing redundant information within the image to suppress noise. Let the noisy image be represented by $\:v$, defined over a discrete, regular grid $\:\varOmega\:\:$of size $\:d$ and cardinality ∣Ω∣. The denoised image $\:u$ at location $\:s\in\:\varOmega\:$ is estimated as:

1 $$\:u\left(s\right)=\:\frac{1}{Z\left(s\right)}\sum\:_{t\in\:\mathcal{N}\left(s\right)}w\left(s,t\right)\:v\left(t\right)\:$$

Here, $\:\mathcal{N}\left(s\right)$ denotes the neighbourhood around $\:s$, $\:w\left(s,t\right)\:$is a similarity-based weight between pixels $\:s$ and $\:t$, and $\:Z\left(s\right)\:$is a normalization factor given by:

2 $$\:Z\left(s\right)=\sum\:_{t\in\:\mathcal{N}\left(s\right)}w\left(s,t\right)\:$$

The weight function $\:w\left(s,t\right)$ measures the similarity between two square patches centred at$\:\:s$ and $\:t,\:$defined as:

3 $$\:w\left(s,t\right)=\:{g}_{h}\left({G}_{\sigma\:}\left(\delta\:\right).\:{\left(v\left(s+\delta\:\right)-v\left(t+\delta\:\right)\right)}^{2}\right)$$

In this equation, Δ denotes the patch region, $\:{G}_{\sigma\:}$ is a Gaussian kernel with variance $\:\sigma\:$, and $\:h$ is a filtering parameter controlling the level of smoothing. The function $\:{g}_{h}\left(x\right)$ can take the form:

4 $$\:{g}_{h}\left(x\right)=\:\frac{1}{1+\left({x}^{2}/{h}^{2}\right)}\:\:$$

5 $$\:{g}_{h}\left(x\right)=\:{e}^{-\left({x}^{2}/{h}^{2}\right)}\:$$

This study uses the former due to its computational efficiency. The algorithm reconstructs the image by taking a weighted average of pixel values, where the weights reflect both intensity similarity and spatial closeness. Equal-sized patches are used to ensure consistent comparison of local structures. Importantly, only pixels within $\:N\left(s\right)$ contribute to the value of $\:u\left(s\right),$ enabling efficient domain decomposition and parallel processing. As per^54^, both the search window N and the patch region Δ are kept of fixed size throughout. The computational complexity of the NLM algorithm is approximately $\:O\left(\left|{\Omega\:}\right|{K}^{d}{P}^{d}\right)$, where K is the size of the search window and P is the patch size. While this complexity grows exponentially with the spatial dimension $\:d$, it remains polynomial with respect to the number of pixels, which is manageable in most practical scenarios. Although expanding the search window $\:N(\cdot\:)$ across the entire image may improve denoising, it also drastically increases computation time. Therefore, consistent with the original NLM framework^54^, this study limits the search to a localized neighborhood. For alternative definitions of neighborhood filtering in the context of NLM, readers can refer to^55^.

## Materials and methods

This study proposes a low-dose CT (LDCT) image denoising technique that integrates a pixel-level non-local self-similarity (NSS) prior with the Non-Local Means (NLM) algorithm. The denoising process is organized into three main stages. In Stage 1, non-local similar pixels are identified to estimate the noise level, followed by the application of the Haar transform with bi-hard thresholding to evaluate local signal intensity and generate a basic denoised image. In Stage 2, the NLM algorithm is applied to refine this preliminary output. Stage 3 involves performing the Haar transform on pixel matrices obtained from both the input image and the NLM-denoised image, applying Wiener filtering, and then executing the inverse Haar transform. Finally, the denoised pixel matrices are aggregated to produce the final restored image. An overview of this complete denoising workflow is illustrated in Fig. 1.

### Stage 1: (a) Search for non-local similar pixels

Let the input noisy image be denoted as $\:y\:\in\:\:{\mathbb{R}}^{h\times\:m}$. The image is divided into a total of N local patches^56^. Each square patch of size $\:\sqrt{n}\times\:\sqrt{n}\:\:$is vectorized into a column vector $\:{y}_{l,1}\:\in\:\:{\mathbb{R}}^{n}$ for $\:l=\text{1,2},\dots\:,\:N$. For each patch $\:{y}_{l,1}$​, its$\:\:m$ most similar patches including itself are identified by computing the Euclidean distance within a sufficiently large search window of size $\:W\times\:W.$ These mmm patches are then stacked column-wise to form the noisy patch matrix:

6 $$\:{Y}_{l}=\:\left[{y}_{l,1},\:{y}_{l,2},\dots\:..,{y}_{l,m}\right]\:\in\:\:\:{\mathbb{R}}^{n\times\:m}$$

To capture pixel-level non-local self-similarity, we analyse each row of $\:{Y}_{l}$​, where each row contains mmm pixels at the same relative location across mmm similar patches. Although patch-level NSS implies some uniformity, variations especially in rare textures or structural details can introduce significant pixel-level discrepancies, leading to artifacts if treated uniformly. To address this, we selectively identify the most consistent rows (i.e., pixels with the highest similarity). For the$\:\:i$-th row $\:{y}_{l}^{i}\in\:\:{\mathbb{R}}^{m}$, the Euclidean distance to the $\:j$-th row $\:{y}_{l}^{i}\left(j=1,\dots\:.,n\right)$ is computed as:

7 $$\:d_{l}^{{\left( {i,j} \right)}} = \:\left\| {y_{l}^{i} - y_{l}^{j} } \right\|_{2} \:$$

Naturally, $\:{d}_{l}^{\left(i,j\right)}$= 0. Based on this metric, we select$\:\:q$ rows (where $\:q$ is a power of 2) that are closest to the reference row $\:{y}_{l}^{i}$​, denoted by:

8 $$\:\left\{{y}_{l}^{\left({i}_{1}\right)},\:{y}_{l}^{\left({i}_{2}\right)},\:\dots\:\dots\:.,\:{y}_{l}^{\left({i}_{q}\right)}\right\}\:,\:\:\:\:\:\:\text{w}\text{h}\text{e}\text{r}\text{e}\:{i}_{1}=i$$

These rows are assembled to form a pixel matrix:

9 $$\:{Y}_{l}^{\left(iq\right)}=\:\left[\left(\begin{array}{ccc}{y}_{l}^{{i}_{1},1}&\:\cdots\:&\:{y}_{l}^{{i}_{1},m}\\\: \vdots &\:\ddots\:&\: \vdots \\\:{y}_{l}^{{i}_{q},1}&\:\cdots\:&\:{y}_{l}^{{i}_{q},m}\end{array}\right)\right]\:\in\:{\mathbb{R}}^{q\times\:m}$$

Here$\:,\:q$ represents the number of similar rows (pixels), and $\:\left\{{i}_{1},\dots\:.,{i}_{q}\right\}\:\subset\:\:\left\{1,\dots\:.,n\right\}\:$. The set of all such matrices $\:\left\{{Y}_{l}^{\left(iq\right)}\right\}\:$for all patches $\:l=1\:,\dots\:,N$ and rows $\:i=1\:,\dots\:,n$ are used in the subsequent steps for accurate noise level estimation.

### Stage 1: (b) noise level Estimation

Accurately estimating the noise level is a crucial step for effective image denoising. We adopt a pixel-level nonlocal self-similarity (NSS) prior for this purpose, which assumes that the selected$\:\:q$ rows from each patch matrix $\:{Y}_{l}^{iq}\in\:\:{R}^{q\times\:m}$ contain highly similar pixels and differ primarily due to additive Gaussian noise. Under this assumption, the statistical variation among these pixels can be attributed to noise, allowing us to compute the standard deviation as an estimate of the noise level. For each row $\:{y}_{li}$​ of matrix $\:{Y}_{l}$​, we identify the $\:q$ most similar rows (including itself), forming the matrix $\:{Y}_{l}^{iq}$​ containing similar pixel values across mmm patches. To quantify the local noise level $\:{\sigma\:}_{l}$, we compute the pairwise Euclidean distances $\:{d}_{l}^{{ii}_{t}}$ between the reference row and its $\:q-1$ most similar rows. Each distance measures the cumulative squared deviation across the mmm corresponding pixel positions.

To convert this cumulative deviation into an average per-pixel estimate, we normalize each squared distance by $\:m$ (the number of similar patches) and then take the square root, yielding the root-mean-square (RMS) deviation. The final noise level $\:{\sigma\:}_{l}$ is then obtained by averaging these RMS deviations across all $\:n$ rows in the patch:

10 $$\:{\sigma\:}_{l}=\frac{1}{n\left(q-1\right)}\sum\:_{t=2}^{q}\sum\:_{i=1}^{n}\sqrt{\frac{1}{m}}{\left({d}_{l}^{{ii}_{t}}\right)}^{2}\:\:\:$$

where $\:\sqrt{1/m}$​ term arises from normalizing the squared Euclidean distance between rows (each consisting of mmm pixels) to obtain a per-pixel root-mean-square (RMS) deviation. This normalization ensures that $\:{\sigma\:}_{l}\:$estimates the standard deviation of noise at the pixel level, rather than over entire patch vectors. This normalization is important for scale-invariant estimation and aligns with the assumption that noise follows a Gaussian distribution. However, we observe that Eq. (5) performs well in smooth regions but may overestimate noise in structured or textured areas where signal and noise are difficult to separate. To address this, we extend the estimation from local to global by computing the average noise level across all$\:\:N$ patch groups:

11 $$\:{\sigma\:}_{g}=\:\frac{1}{N}\sum\:_{l=1}^{N}{\sigma\:}_{l}\:$$

This global noise level $\:{\sigma\:}_{g}$​ is then used in the subsequent denoising stages to ensure consistency and robustness across varying image regions.

The use of a Gaussian model for noise and the pixel-wise NSS assumption is supported by prior studies^57^, and the formulation in Eq. (10) offers a computationally simple yet accurate means of estimating noise levels directly from the image data.

### Stage 1: (c) Estimation of local signal intensity using Haar transform-based bi-thresholding

In this step, we estimate the local signal intensity from the grouped matrices $\:{Y}_{l}^{q}\in\:\:{\mathbb{R}}^{q\times\:m}$, where each matrix consists of pixels with similar intensity patterns across non-local patches for each $\:l=1,...,N$. For simplicity, the pixel index$\:\:i$ is omitted. A global noise level $\:\sigma\:$ is first estimated, and the denoising is then performed in the Haar transform domain^58^. To accomplish this, we employ the lifting Haar wavelet transform (LHWT)^59^, which is chosen for its computational efficiency, low memory footprint, and adaptability. LHWT is applied to each noisy pixel matrix using two orthogonal transform matrices, $\:{H}_{l}\:\in\:{\mathbb{R}}^{q\times\:\text{q}}$ and $\:{H}_{r}\:\in\:{\mathbb{R}}^{m\times\:\text{m}}$, where both$\:\:q$ and $\:m\:$are powers of two. The transformation yields a coefficient matrix $\:{C}_{l}^{q}\:\in\:{\mathbb{R}}^{q\times\:\text{m}}$given by:

12 $$\:{C}_{l}^{q}={H}_{l\:}.\:\:{Y}_{l}^{q}{\:.\:\:H}_{r}$$

To suppress noise, we apply element-wise hard thresholding to each coefficient $\:{C}_{l}^{q}\left(i,j\right)$ by retaining only those values exceeding a predefined threshold $\:\tau\:{\sigma\:}_{g}^{2}$. The resulting thresholded matrix $\:{\widehat{C}}_{l}^{q}$​ is computed as:

13 $$\:{\widehat{C}}_{l}^{q}=\:{C}_{l}^{q}\circ\:{I}_{\left|{C}_{l}^{q}\right|\:\ge\:\:\tau\:{\sigma\:}_{g}^{2}}\:,\:$$

Here, ∘ denotes element-wise multiplication and $\:I$ is the indicator function. According to wavelet theory^59^, the final two rows of $\:{C}_{l}^{q}$ (excluding the first column) primarily capture high-frequency components, which often correspond to noise. To further suppress these components, we apply structural hard thresholding by zeroing out all coefficients in these rows, producing the refined matrix $\:{\stackrel{-}{C}}_{l}^{q}$

14 $$\:{\stackrel{-}{C}}_{l}^{q}\left(i,j\right)={\widehat{C}}_{l}^{q}\left(i,j\right)\:\circ\:\:{I}_{if\:i=1,\dots\:..,q-2\:or\:j=1}\:\:$$

The denoised pixel matrix $\:{\stackrel{-}{Y}}_{l}^{q}$​ is then reconstructed from the thresholded coefficients by applying the inverse LHWT:

15 $$\:{\stackrel{-}{Y}}_{l}^{q}=\:{H}_{il}\:.\:{\stackrel{-}{C}}_{l}^{q}\left(i,j\right)\:.\:{H}_{ir,}\:\:$$

where $\:{H}_{il}\in\:{\mathbb{R}}^{q\times\:\text{q}}$ and $\:{H}_{ir}\in\:{\mathbb{R}}^{m\times\:\text{m}}\:$are the corresponding inverse LHWT matrices. The details of both forward and inverse LHWT operations for specific values of $\:q$ and $\:m$ are available in^56^. Finally, all denoised matrices $\:{\stackrel{-}{Y}}_{l}^{q}$ are aggregated to reconstruct the globally denoised image. The refined coefficients $\:{\stackrel{-}{C}}_{l}^{q}\:$represent local signal intensities, which are critical for accurate image restoration. To ensure optimal estimation of these intensities, the LHWT-based bi-hard thresholding is iteratively applied for K cycles.

### Stage 2: fast non-local means algorithm

The central objective of this stage is to efficiently compute the similarity weights $\:w(i,j$) as defined in Eq. (2), which are critical for the denoising process. To simplify the explanation, we consider a one-dimensional (1D) case, which can be easily extended to higher dimensions. Let the 1D image domain be $\:{\Omega\:}=\left\{\text{0,1},\dots\:..,n-1\right\}$, where $\:n$ is the total number of pixels. Given a translation vector $\:{d}_{x}$​, we define a function $\:{S}_{{d}_{x}}\left(p\right)$ to compute the cumulative squared difference between the denoised image $\:{\stackrel{-}{Y}}_{l}^{q}\:$and its shifted version:

16 $$\:{S}_{{d}_{x}}\left(p\right)=\sum\:_{k=0}^{p}{\left({\stackrel{-}{Y}}_{l}^{q}\left(k\right)-{\stackrel{-}{Y}}_{l}^{q}\left(k+{d}_{x}\right)\right)}^{2}\:\:\:\:\:\:p\in\:{\Omega\:}\:$$

This function provides a pre-integrated difference measure that significantly accelerates the computation of similarity weights. Since calculating $\:{S}_{{d}_{x}}\left(p\right)$ requires accessing pixels beyond the image boundaries, symmetric or periodic padding is employed to preserve data consistency and prevent cache overflow during processing. In the 1D scenario, the patch window is defined as $\:\varDelta\:= \left[\kern-0.15em\left[ { - P, \ldots \:.,P} \right]\kern-0.15em\right]$. To reduce computational complexity, we replace the Gaussian kernel traditionally used for weighting with a constant value, assuming negligible variation. With this simplification, Eq. (2) becomes:

17 $$\:\text{w}\left(i,j\right)={g}_{h}\left(\sum\:_{{{\updelta\:}}_{x}\in\:{\Delta\:}}{\left({\stackrel{-}{Y}}_{l}^{q}\left(i+{\delta\:}_{x}\right)-{\stackrel{-}{Y}}_{l}^{q}\left(j+{\delta\:}_{x}\right)\right)}^{2}\right)$$

Here, $\:{g}_{h}\:$​(⋅) is a non-increasing function that quantifies the similarity between two-pixel neighbourhoods. To optimize further, we set $\:{d}_{x}=\:j-i$ and $\:\widehat{p}=i+{{\updelta\:}}_{x}$​, enabling a reformulation of the weight expression:

18 $$\:\text{w}\left(i,j\right)={g}_{h\:}\left(\sum\:_{\widehat{p}=i-\mathsf{P}}^{i+\mathsf{P}}{\left({\stackrel{-}{Y}}_{l}^{q}\left(\widehat{p}\right)-{\stackrel{-}{Y}}_{l}^{q}\left(\widehat{p}+{d}_{x}\right)\right)}^{2}\right)\:$$

This formulation allows us to compute weights more efficiently by referencing the precomputed $\:{S}_{{d}_{x}}$values:

19 $$\:\stackrel{-}{w}\left(i,j\right)={g}_{h}\left({S}_{{d}_{x}}\left(i+\mathsf{P}\right)-{S}_{{d}_{x}}\left(j-\mathsf{P}\right)\right)\:$$

Since $\:{S}_{{d}_{x}}\:$is known and P is fixed, this computation becomes independent of the patch size, significantly reducing complexity. This key result enables fast and scalable similarity evaluation across large image domains. In general, for $\:d$-dimensional images, the approach results in a computational complexity of $\:O\left({2}^{d}\right)\:,\:$making it highly efficient compared to conventional formulations, such as that in^54^, which scale with both patch size and image dimensions. The fast NLM algorithm proceeds as follows: first, all $\:{S}_{{d}_{x}}\:$values are computed using Eq. (16); next, the similarity weights are calculated using Eq. (2) through (17); finally, denoising is performed by weighted averaging based on Eq. (1). This process is repeated across all translation vectors within the defined search window.

### Stage 3: Wiener filtering for final denoising

The denoised image obtained from Stage 2 serves as a preliminary estimate of the clean image but may still retain residual noise. To refine this result, Stage 3 applies Wiener filtering for enhanced denoising while preserving important structural details. This process leverages soft-thresholding within the Haar transform domain, guided by the previously estimated local signal intensities and global noise level $\:{\sigma\:}_{g}$​. Wiener filtering is applied to the LHWT (Lifting Haar Wavelet Transform) coefficients of the original noisy pixel matrices. The filter adaptively attenuates noise based on local signal-to-noise characteristics. Specifically, for each coefficient $\:{C}_{l}^{q}\left(i,j\right)$ from Eq. (12), the Wiener-filtered output $\:{\stackrel{-}{C}}_{l}^{q}\left(i,j\right)$ is computed as:

20 $$\:{\stackrel{-}{C}}_{l}^{q}\left(i,j\right)=\:\frac{{\left|\stackrel{-}{w}\left(i,j\right)\right|}^{2}}{{\left|\stackrel{-}{w}\left(i,j\right)\right|}^{2}+{\left({\sigma\:}_{g}/2\right)}^{2}}\:\:{C}_{l}^{q}\left(i,j\right)\:$$

Here, $\:\stackrel{-}{w}\left(i,j\right)\:$represents the pixel value obtained from the denoised output of Stage 2 (Eq. 19), and $\:{\sigma\:}_{g}$ denotes the global noise level. Notably, this stage requires both the original noisy image and its denoised counterpart from Stage 2 to effectively compute the filtering coefficients. To apply the Wiener filter, the matrices of non-local similar pixels from both the input and the denoised image are first transformed using LHWT. Wiener filtering is then applied in the transform domain using Eq. (20), and the filtered coefficients are subsequently transformed back into the spatial domain via inverse LHWT, yielding the refined denoised matrix $\:{\stackrel{-}{Y}}_{l}^{q}$​. Finally, all such filtered matrices $\:{\stackrel{-}{Y}}_{l}^{q}\:$are aggregated across the image to produce the final, high-quality denoised output. This stage enhances detail preservation and significantly reduces remaining noise offering a more accurate restoration of the latent clean image.

**Figure:**
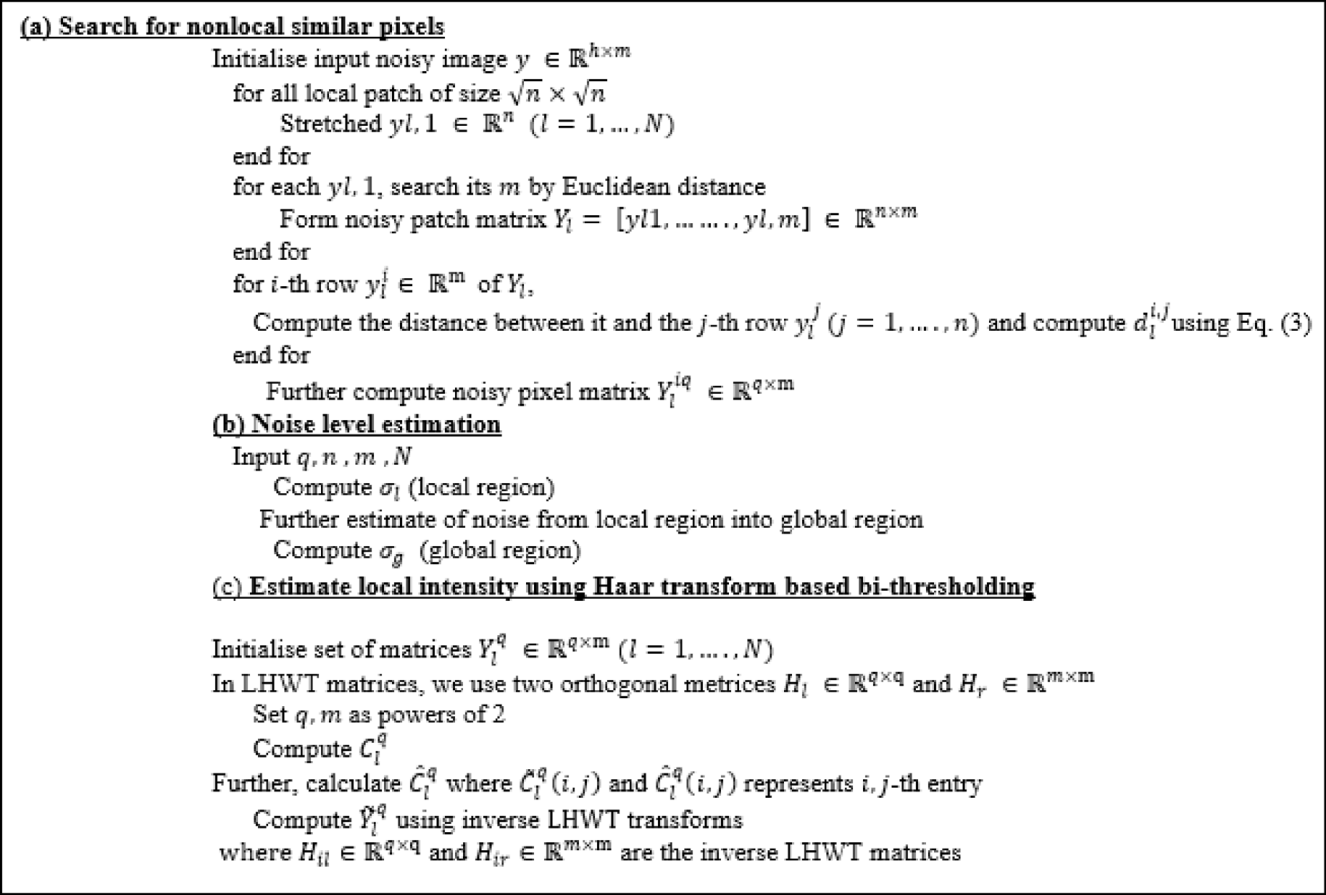
Algorithm 1. Pseudocode for Stage 1.

**Figure:**
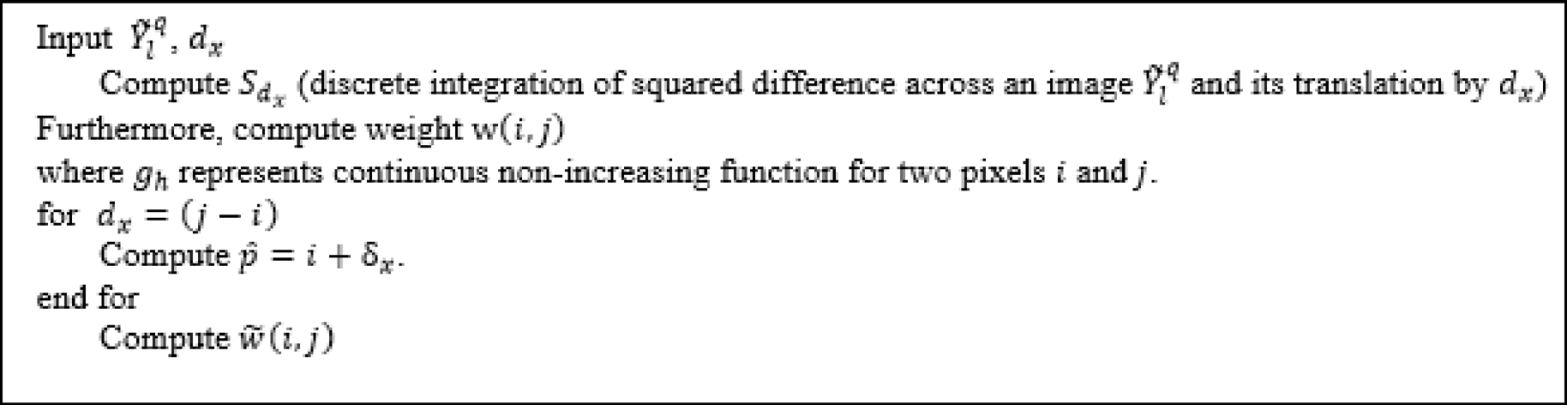
Algorithm 2. Pseudocode for Stage 2.

**Figure:**
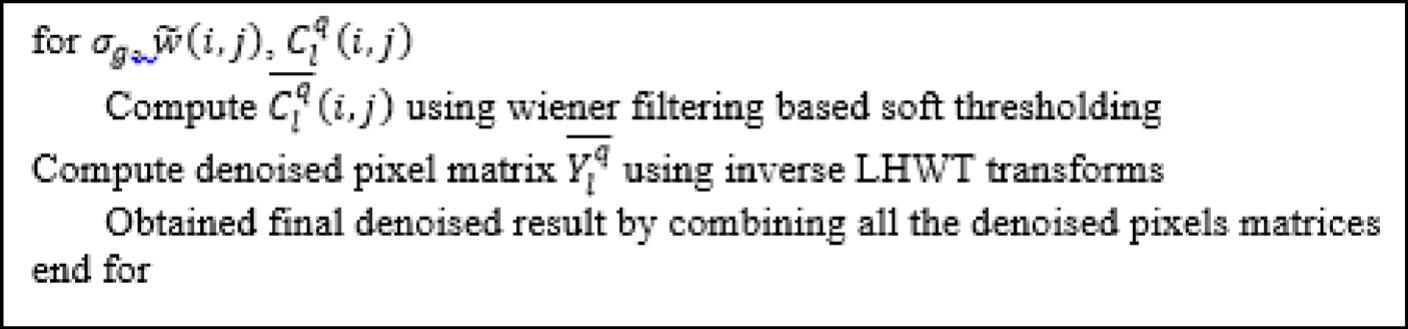
Algorithm 3. Pseudocode for Stage 3.

## Experimental results and analysis

In this study, we utilized the publicly available LDCT dataset from the 2016 NIH-AAPM-Mayo Clinic Low-Dose CT Grand Challenge^60^ which comprises a total of 2378 slices of 3 mm full-dose CT images from 10 patients^61,62^. The dataset was acquired using a Siemens 16-slice multidetector CT scanner operating at a tube voltage of 120 kVp. To ensure robust training and reproducibility of our model, we partitioned the data such as 780 images from seven patients were randomly selected for training, 140 images from one patient were used as a validation set, and 220 images from the remaining two patients for testing. This patient-wise split was implemented to evaluate the generalizability of the model to unseen patient data. In addition, for detailed qualitative and quantitative evaluation, we included representative results from four specific patients: N284 (Head CT), N056 (Head CT), L147 (Abdomen CT), and L082 (Abdomen CT) for representation in the manuscript. This diverse selection further demonstrates the effectiveness of our method across different anatomical regions supporting its clinical applicability and reproducibility. In this study, low-dose CT (LDCT) images were simulated by introducing Gaussian noise into sinograms generated from normal-dose CT (NDCT) images. Gaussian noise with varying intensities, corresponding to standard deviation ($\:\sigma\:$) values ranging from 40 to 5, was added to simulate different radiation dose levels while maintaining control over the noise characteristics. This approach enables a comprehensive evaluation of the proposed denoising method across a range of noise scenarios. The simulated LDCT images exhibit prominent streak artifacts and noise, especially near regions with high attenuation, such as bone structures. The original NDCT images serve as the ground truth for assessing denoising performance using quantitative metrics. To benchmark the effectiveness of the proposed method, we compared it against several state-of-the-art denoising techniques, including Wasserstein distance and perceptual loss-based GAN (WDPL-GAN)^17^, residual convolutional network with fractional total variation loss (RCN-FTV)^4^, weighted coding with sparse non-local regularization (WC-SNR)^5^, task-oriented denoising network (TOD-Net)^23^, and wavelet sub-band-specific learning (WSL)^33^. The source codes for these methods were obtained from the respective authors’ official repositories and executed using their default parameter configurations. These baseline models were implemented in Python using the TensorFlow framework and run on an NVIDIA Titan XP GPU. In contrast, all experiments for the proposed method were conducted using MATLAB on an Intel(R) Core (TM) i9-9900 K CPU @ 3.60 GHz with 16 GB of RAM.

### Parameters settings

**Fig. 2:**
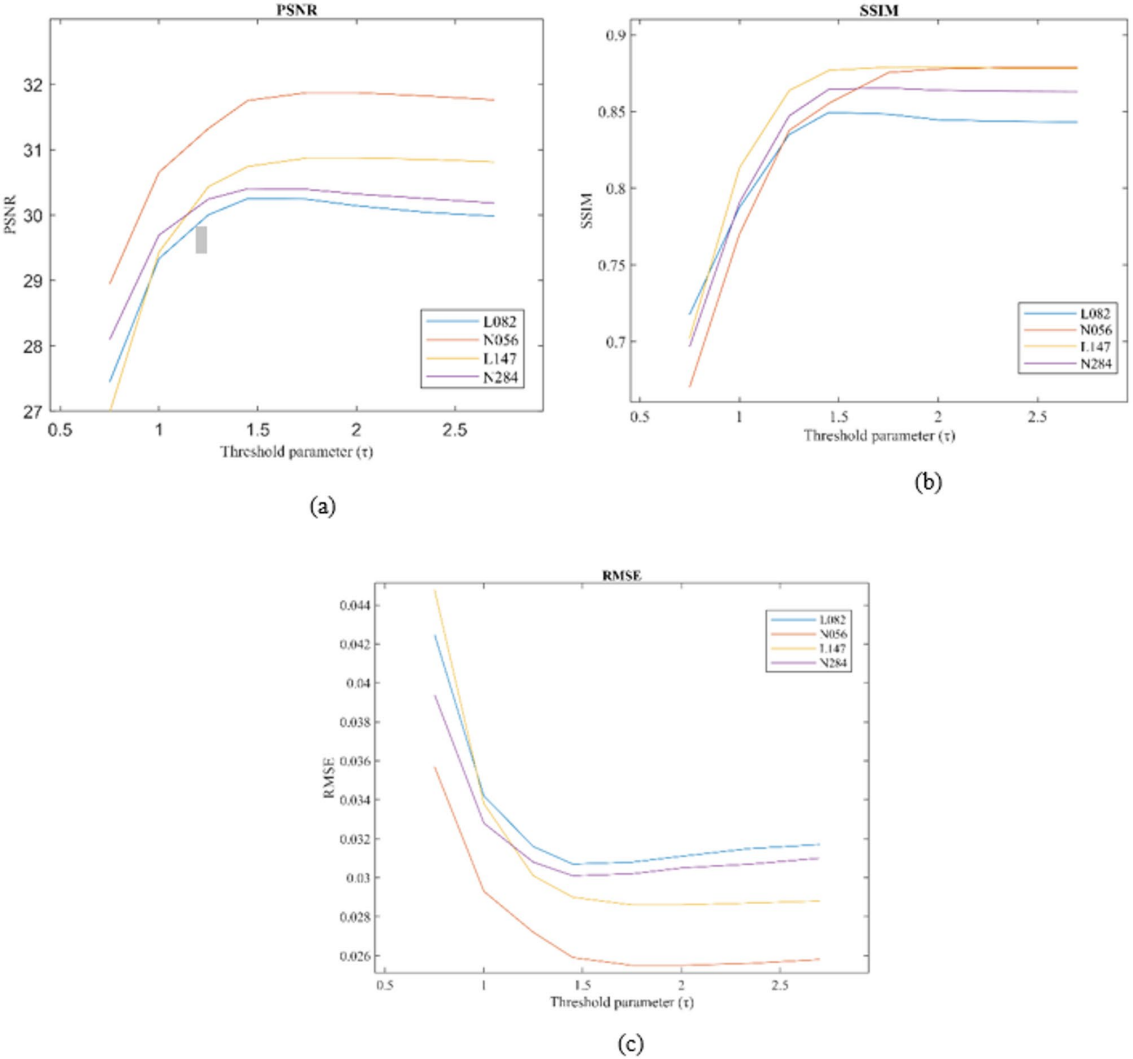
Performance of the proposed method with respect to PSNR, SSIM, and RMSE across different values of the threshold parameter ($\:\tau\:$).

For the existing LDCT image denoising methods, including WDPL-GAN, WC-SNR, TOD-Net, RCN-FTV, and WSL, the parameters were configured as specified by the respective authors in their original publications. For the proposed algorithm, the parameters were set as follows: $\:P=5,\:K=3,\:and\:h=0.15$. In addition, several key parameters were consistently used throughout all experiments such as the regularization parameter λ = 0.6, patch size $\:\sqrt{n}$​, hard threshold parameter$\:\:\tau\:$ = 2, number of similar pixels $\:q\:$= 4, window size $\:W$ = 40 for searching similar patches, number of iterations $\:K$, and number of similar patches $\:m$ = 16. Figure 2 shows performance of the proposed method (w.r.t PSNR, SSIM and RMSE) in terms of threshold parameter (*τ).*

### Qualitative evaluation

**Fig. 3:**
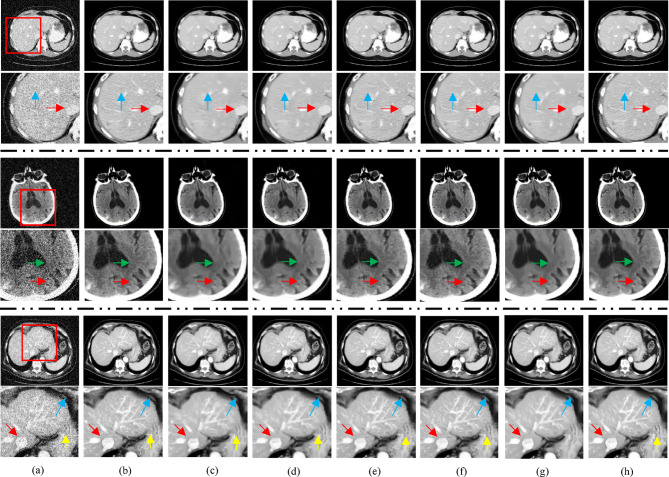
Qualitative comparison of transverse CT slices for three cases: L082 (Top), N056 (Middle), and L147 (Bottom) processed using different denoising algorithms. The corresponding results are shown for: (**a**) LDCT input, (**b**) NDCT ground truth, (**c**) WDPL-GAN, (**d**) WC-SNR, (**e**) TOD-Net, (**f**) RCN-FTV, (**g**) WSL, and (**h**) the proposed method. Zoomed regions of interest (ROIs), highlighted by red squares in the original images are shown below in enlarged views for detailed evaluation. Red arrows indicate white edge artifacts, blue arrows highlight preserved edges, and green and yellow arrows point to small structural details.

**Fig. 4:**
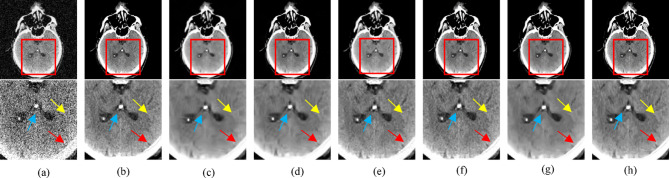
Qualitative comparison of transverse CT image N284 processed using various denoising algorithms: (**a**) LDCT input, (**b**) NDCT ground truth, (**c**) WDPL-GAN, (**d**) WC-SNR, (**e**) TOD-Net, (**f**) RCN-FTV, (**g**) WSL, and (**h**) the proposed method. Enlarged views of the red-marked regions of interest (ROIs) are presented below each image for detailed visual inspection. Blue and red arrows indicate bone structures, while yellow arrows highlight smaller anatomical features.

To demonstrate the effectiveness of the proposed method in preserving fine structural details during denoising, we performed a visual comparison using selected CT images. These images include cases with low-attenuation lesions as well as regions severely affected by artifacts scenarios that are critical for clinical evaluation. The qualitative results, shown in Figs. 3 and 4, highlight the capability of our approach to suppress noise while retaining diagnostically relevant features. The “L082”, “N056”, “L147”, and “N284” refer to specific selected images from the NIH-AAPM-Mayo Clinic Low-Dose CT Grand Challenge dataset, which is a standard benchmark used for image denoising performance evaluation. These identifiers are image file names used within the dataset. The red squares in Figs. 3 and 4 highlight zoomed regions of interest (ROIs) for detailed visual assessment. While all compared methods achieved some level of denoising, the low-dose CT (LDCT) images in Figs. 3b and 4b exhibit significant degradation due to reduced photon counts from the incident X-ray, resulting in poor visibility of structural details. Although all algorithms shown in Figs. 3 and 4 reduced noise to a certain extent, residual noise and artifacts remain visible particularly in Figs. 3c and 4c. WC-SNR, as shown in Figs. 3d and 4d, performed slightly better than WDPL-GAN in terms of noise suppression. However, it introduces blocky artifacts and tends to blur fine edges and small structures.

More recent methods demonstrate improved performance, outperforming both WDPL-GAN and WC-SNR by more effectively suppressing noise and removing artifacts. Nevertheless, differences in noise reduction persist, as seen in Figs. 3g and 4g. For instance, WSL excessively smooths the denoised images, leading to the loss of important anatomical features and blurring of clinically significant regions. Deep learning-based methods generally preserve structural details more effectively while reducing noise and artifacts. Notably, our proposed method, as illustrated in Figs. 3h and 4h, achieves superior performance by retaining more content-rich information and finer textural details. This leads to improved visual clarity, which is essential for diagnostic accuracy. For example, metastases outlined with red dashed squares in Figs. 3f and 4f remain difficult to detect with some methods, whereas our method produces sharper and more distinguishable results. Compared to other techniques, our method clearly enhances the visibility of subtle features such as the bright spots indicated by blue arrows with minimal surrounding distortion. To further demonstrate the advantage of our approach, the absolute difference images between the denoised outputs and the ground truth (NDCT) are also presented in Figs. 3 and 4. These visual comparisons confirm that our proposed method is more effective in reducing artifacts and noise, yielding final outputs that closely resemble the corresponding normal-dose CT images.

### Quantitative evaluation

To assess denoising performance quantitatively, we employed three widely used evaluation metrics: Peak Signal-to-Noise Ratio (PSNR)^63^, Structural Similarity Index Measure (SSIM)^64^, and Root Mean Square Error (RMSE)^65^. PSNR and RMSE evaluate pixel-level fidelity, while SSIM measures structural similarity within localized image regions. The results of these metrics for all evaluated methods are summarized in Tables 1 and 2. Although methods like WC-SNR and WSL achieve relatively high PSNR and low RMSE scores, their visual outputs (Figs. 3 and 4) reveal a tendency to over-smooth the images, which leads to the loss of fine structural details. This excessive smoothing often results in lower SSIM values, indicating diminished structural preservation. In contrast, our proposed method demonstrates superior performance in both SSIM and visual quality, particularly when compared to RCN-FTV and WSL. It achieves higher PSNR and lower RMSE, while maintaining greater structural fidelity, which is essential for accurate clinical interpretation.

**Table 1:** Quantitative comparison of various denoising algorithms on the simulated test set (L082, N056, L147). The best results are shown in bold, and the second-best results are *underlined*.

Method	L082			N056			L147		
	PSNR ($\:\uparrow\:)$	SSIM ($\:\uparrow\:)$	RMSE $\:(\downarrow\:)$	PSNR ($\:\uparrow\:)$	SSIM ($\:\uparrow\:)$	RMSE$\:(\downarrow\:)$	PSNR ($\:\uparrow\:)$	SSIM ($\:\uparrow\:)$	RMSE$\:(\downarrow\:)$
LDCT	25.7762	0.7852	0.0465	26.9862	0.8142	0.0356	25.8992	0.8072	0.0373
WDPL-GAN^63^	26.7721	0.8054	0.0433	28.1442	0.8342	0.0345	26.8772	0.8134	0.0364
WC-SNR^5^	27.4551	0.8155	0.0425	28.5624	0.8543	0.0333	27.1662	0.8221	0.0355
TOD-Net^23^	28.4562	0.8266	0.0409	29.7725	0.8621	0.0327	27.8827	0.8267	0.0345
RCN-FTV^4^	29.1231	0.8332	*00355*	30.1233	0.8655	*0.0282*	*29.7761*	0.8378	0.0324
WSL^33^	*29.5624*	*0.8382*	0.0367	*30.4552*	*0.8734*	0.0285	29.7726	*0.8443*	*0.0315*
Ours	**30.2848**	**0.8424**	**0.0306**	**31.8535**	**0.8854**	**0.0255**	**30.6971**	**0.8562**	**0.0292**

**Table 2:** Quantitative performance comparison of various denoising algorithms on the simulated test case N284. The highest-performing results are highlighted in bold, while the second-best results are *underlined*.

Method	N284		
	PSNR ($\:\uparrow\:)$	SSIM ($\:\uparrow\:)$	RMSE$\:(\downarrow\:)$
LDCT	24.2314	0.8167	0.0373
WDPL-GAN^63^	25.6788	0.8333	0.0366
WC-SNR^5^	26.6277	0.8467	0.0356
TOD-Net^23^	27.8826	0.8536	0.0351
RCN-FTV^4^	28.8827	*0.8654*	0.0343
WSL^33^	*29.5661*	0.8644	*0.0324*
Ours	**30.3960**	**0.8761**	**0.0302**

**Fig. 5:**
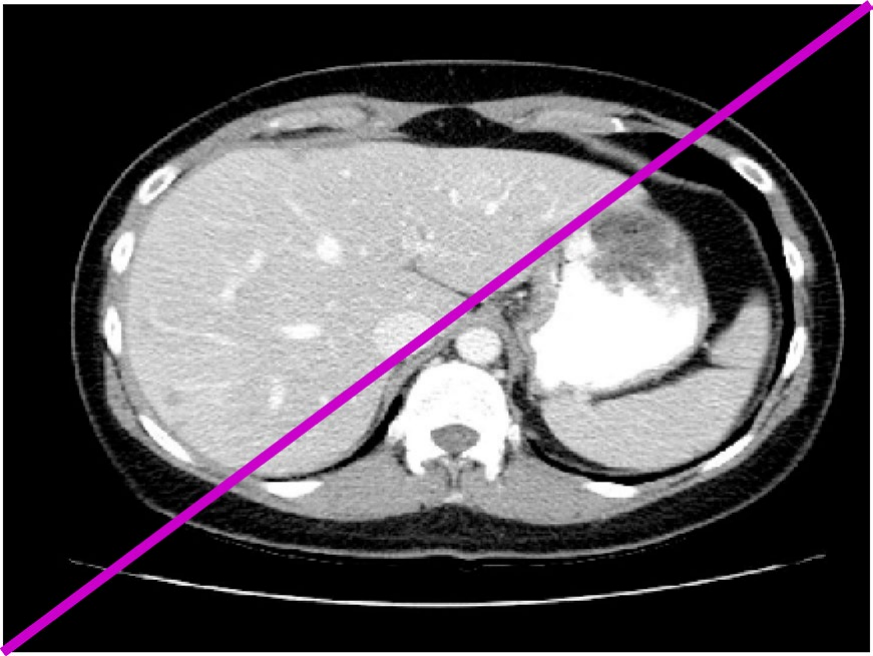
Reference line used for intensity profile analysis in image L082 across all evaluated methods.

**Fig. 6:**
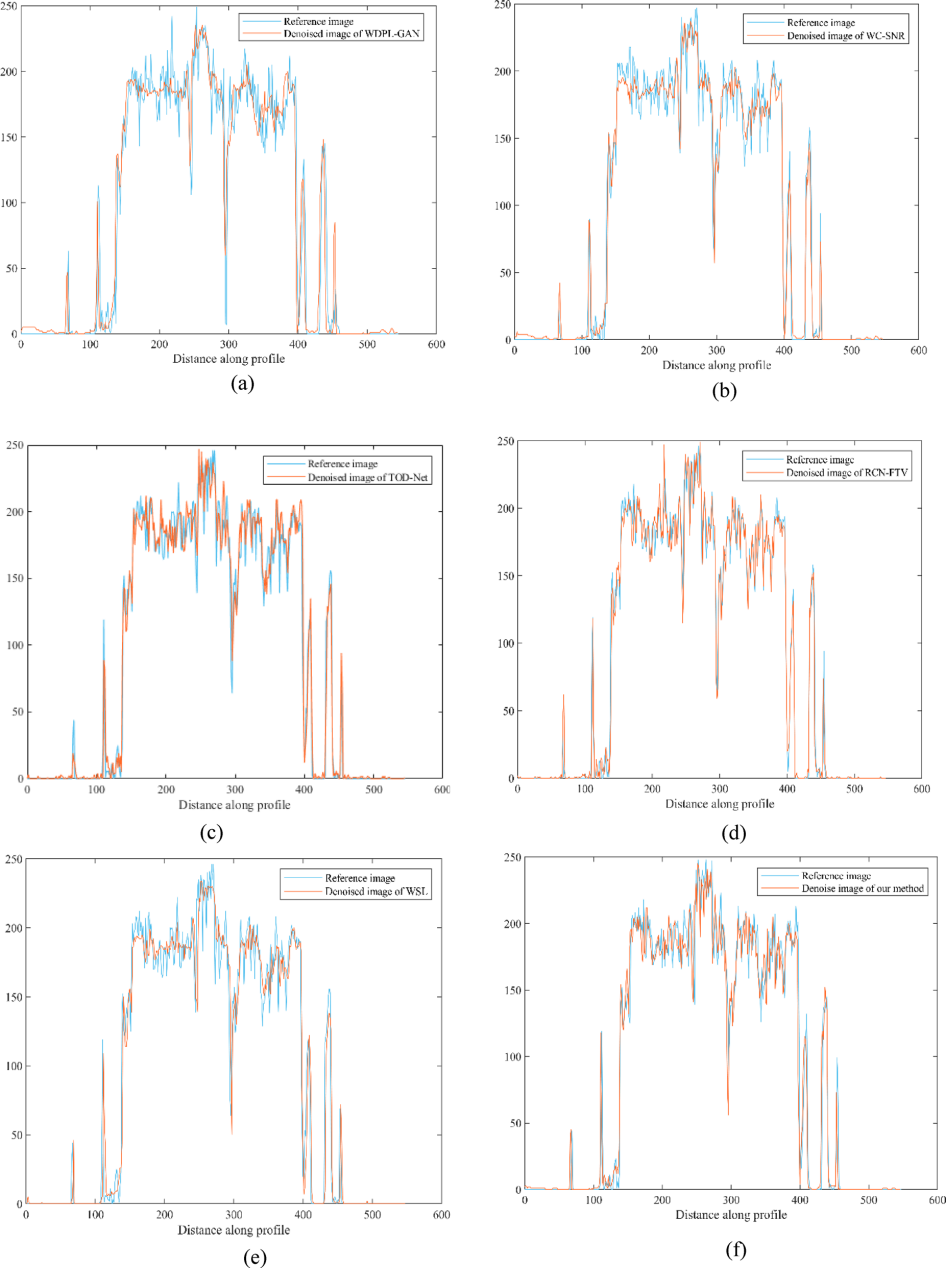
Intensity profile comparison for the L082 image across different denoising methods: WDPL-GAN, WC-SNR, TOD-Net, RCN-FTV, WSL, and the proposed method. The original NDCT image is included as a reference for evaluating the preservation of structural detail and noise suppression.

**Fig. 7:**
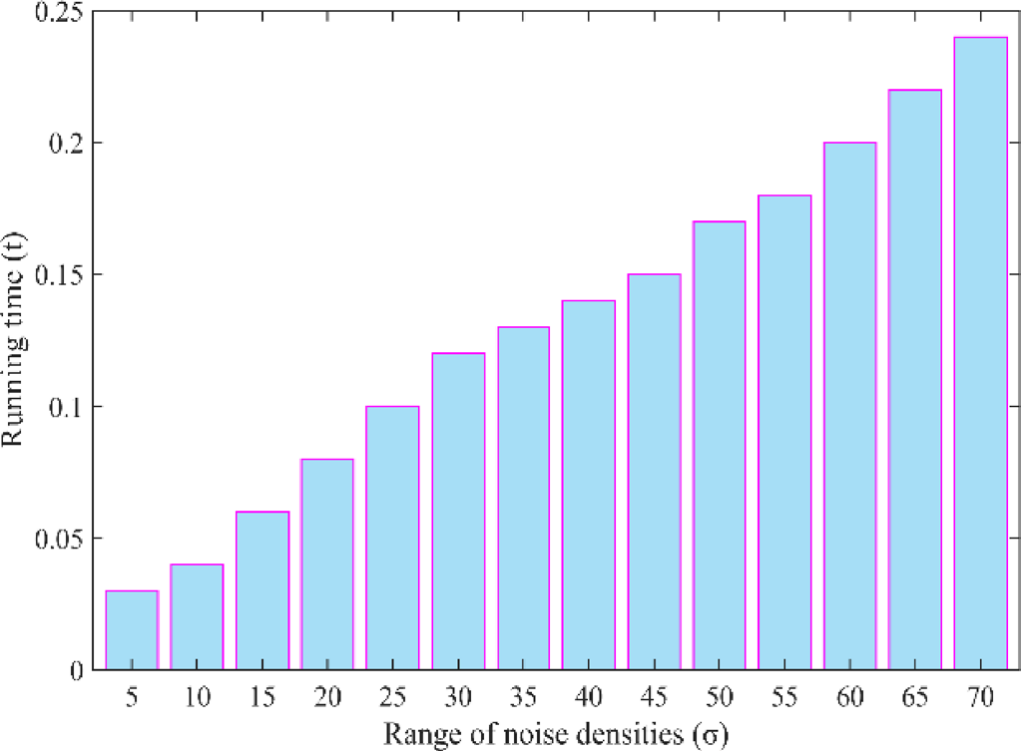
Running time of proposed method w.r.t range of noise densities.

The RCN-FTV method preserves a significant amount of structural information, which is critical for diagnostic analysis; however, this comes at the cost of lower performance in quantitative evaluation metrics such as RMSE and PSNR. It is important to note that both RMSE and PSNR are pixel-level metrics and do not always correlate well with human perceptual assessment of image quality. Figure 2 presents the variation of SSIM, PSNR, and RMSE values for our method as a function of the threshold parameter (τ), evaluated across four selected CT images. Among these, cases N056 and L147 achieved consistently better results across all metrics, whereas L082 showed comparatively lower performance. Ideally, denoising algorithms should reconstruct latent clean CT images whose statistical characteristics closely resemble those of their corresponding normal-dose (NDCT) counterparts. The WDPL-GAN method achieved average metric values that aligned with its visual outputs observed in Figs. 3 and 4. With the exception of entropy, our proposed method achieved the best overall performance in Tables 1 and 2. While WC-SNR tended to produce overly smoothed images, TOD-Net showed limited effectiveness in suppressing noise in LDCT images. Notably, the statistical scores achieved by our method across all test images were closest to those of the NDCT reference images. This similarity in textural and statistical features demonstrates our method’s effectiveness in reconstructing high-fidelity denoised images. The primary objective of this study was to develop an LDCT denoising approach capable of producing results that are as visually and quantitatively comparable to NDCT images as possible. To further support this analysis, Fig. 5 illustrates the reference line used for intensity profile evaluation, and Fig. 6 presents the corresponding intensity profiles comparing our method with WDPL-GAN, RCN-FTV, WC-SNR, TOD-Net, and WSL. Although all frameworks showed some level of denoising performance, WDPL-GAN and WC-SNR introduced noticeable blurring and over-smoothing artifacts due to excessive correction during the denoising process. Our method was developed to effectively address the challenges of LDCT denoising and was benchmarked against both traditional and deep learning-based state-of-the-art approaches. Denoising LDCT images is particularly challenging due to the complex, structured noise introduced during image reconstruction. While methods like WDPL-GAN and RCN-FTV are designed for specific types of noise, they are less suited for the noise patterns typical in LDCT data and thus fail to provide satisfactory results in this context. Compared to these conventional techniques, deep learning-based methods offer more powerful representation capabilities due to their ability to model complex functions and extract high-level features from training data. This allows them to deliver impressive denoising results. However, deep learning models also come with significant drawbacks, including high computational requirements, memory consumption, and the.

need for large datasets for effective training and testing. In contrast, our proposed method is computationally efficient, easy to implement, and produces superior results both visually and quantitatively. It achieves excellent noise suppression and artifact removal without the need for extensive resources. While WSL reported low RMSE and high PSNR scores, it suffered from over-smoothing, resulting in images that appear unnatural and blurry. Though such methods can suppress background noise, they often ignore the local structural variations that are essential in LDCT images. In reality, noise in LDCT images reconstructed via filtered back projection (FBP) is spatially structured and correlated with anatomical features. Thus, our approach is well-suited for LDCT denoising, offering an optimal balance between performance and computational efficiency. It outperforms other methods evaluated in this study in terms of preserving image detail, suppressing noise, and maintaining fidelity to standard-dose CT images. The influence of the running time at several range of noise densities has been demonstrated in Fig. 7.

## Discussion

This study highlights the importance of low-dose CT (LDCT) image denoising in reducing unwanted noise and streak artifacts, which can otherwise compromise diagnostic accuracy. For evaluation, we used a set of images from a standard benchmark dataset^60^. Both the normal-dose CT (NDCT) and the corresponding simulated LDCT images are shown in Figs. 3 and 4. To facilitate detailed comparisons, regions of interest (ROIs) have been highlighted using red boxes and circles in these figures. The LDCT images clearly exhibit streak artifacts and mottling noise, particularly in regions with low contrast. Our proposed method significantly improves image quality by effectively suppressing these artifacts while preserving structural details. In contrast, denoised results produced by the WDPL-GAN algorithm still contain visible noise and artifacts. Visual analysis reveals that WC-SNR outputs suffer from noticeable blocky effects, while TOD-Net, RCN-FTV, and WSL leave residual noise and artifacts in various degrees. Among these, RCN-FTV performs better than TOD-Net, particularly in flatter regions, but still fails to fully suppress noise. Figure 2 illustrates the.

performance of our method in terms of SSIM, PSNR, and RMSE with varying amplitude threshold (τ). Notably, our method consistently achieves higher SSIM and PSNR scores compared to competing techniques, while maintaining a lower RMSE which is ideal for effective denoising. These metrics confirm the superiority of our method over existing techniques, including WDPL-GAN, WC-SNR, TOD-Net, RCN-FTV, and WSL. The WSL algorithm, despite achieving reasonable RMSE and PSNR values, still suffers from residual noise and tends to over-smooth structural details. In contrast, our method demonstrates a strong ability to eliminate both noise and artifacts while retaining anatomical features critical for clinical interpretation. Overall, our method outperforms the state-of-the-art approaches in terms of visual quality and quantitative metrics. In all test cases, it yields higher PSNR and SSIM values and consistently achieves the lowest RMSE. Furthermore, our method performs exceptionally well in ROI analysis, producing cleaner and more consistent outputs compared to other algorithms. The input LDCT images, characterized by heavy noise and streak artifacts, were significantly improved by our technique. While WDPL-GAN effectively reduces artifacts, its results often show blocky distortions, especially in uniform areas. WC-SNR, although good at preserving edges and structural details, retains mottling noise and fails to remove all streak artifacts. Among all evaluated approaches, our method delivers the most balanced and reliable performance. The metrics SSIM, PSNR, and RMSE were used for objective evaluation of the denoised images. While deep learning-based methods often show strong performance, they also demand significantly higher computational resources and longer processing times during both training and testing. In contrast, our method requires substantially less computational time and memory, making it more efficient and practical for clinical use.

The pixel-level nonlocal self-similarity (NSS) information has been extensively utilized in neural networks such as non-local neural networks and transformer-based architectures, which leverage self-attention mechanisms to capture relationships between non-local regions in an image. These methods typically operate by modelling similarities at a patch or feature level, which is computationally intensive and less precise when the goal is to exploit fine-grained pixel relationships. In contrast, the proposed method focuses directly on a pixel-level NSS prior by searching for closely similar pixels across a non-local region. This approach is motivated by the observation that identifying similar pixels in natural images is more feasible and effective than comparing entire patches, especially in images with high-frequency details or complex textures. By emphasizing pixel-level NSS, the proposed method avoids the redundancy of patch-based processing and directly enhances the precision of the denoising process. This technique not only reduces computational complexity but also improves the adaptability and effectiveness of image denoising models, making it a promising alternative to patch-based NSS methods used in traditional and transformer-based neural networks. The proposed method focuses on searching for closely similar pixels within a non-local region, rather than larger patches to enhance image denoising. Searching for similar pixels is computationally less expensive and more feasible than patch-based approaches, especially in natural images where exact patch matches are limited. The method can enhance fine-grained details and effectively reduce noise while preserving edges and textures by focusing on pixel-level NSS. Only limitation would be pixel-level NSS may struggle with extremely noisy images were identifying closely similar pixels.

## Conclusion

This study presents an efficient denoising method for low-dose CT (LDCT) images, leveraging a pixel-level nonlocal self-similarity (NSS) prior combined with a nonlocal means algorithm. Unlike traditional patch-based approaches, this method enhances denoising performance by extending the NSS concept to the pixel level enabling a more precise estimation of noise levels. These estimates are then utilized to construct a robust LDCT image denoising algorithm. The technique involves computing local signal intensity using a non-local Haar transform with bi-hard thresholding, followed by denoising through Wiener filtering-based soft thresholding. To improve computational efficiency, a fast approach for non-local filtering was also proposed. The denoising process employs an optimized version of the nonlocal means filter that delivers superior performance. Furthermore, the implementation benefits from single instruction multiple data (SIMD) capabilities, which allow for faster and more scalable processing on modern memory-intensive computing platforms through vectorized and parallel execution. Extensive experiments were conducted on a publicly available benchmark dataset. The proposed method demonstrated significant improvements over existing techniques, particularly in terms of noise suppression and artifact reduction. This study highlights promising directions for future research including ongoing work to adapt the proposed method for use across various CT scanners to further enhance denoising outcomes.

## Data Availability

The dataset utilized in this study is publicly available through the official website of the 2016 NIH-AAPM Low-Dose CT Grand Challenge, hosted by the Mayo Clinic: https://www.aapm.org/GrandChallenge/LowDoseCT/.

## References

[1] Computed Tomography. Principles, design, artifacts, and recent Advances-Jiang Hsieh-Google books. (accessed 01 Aug 2024).

[2] Shangguan, H. et al. Low-dose CT statistical iterative reconstruction via modified MRF regularization. *Comput. Methods Progr. Biomed.* **123**, 129–141. 10.1016/J.CMPB.2015.10.004 (2016). 10.1016/j.cmpb.2015.10.004 26542474

[3] Kalra, M. K. et al. Strategies for CT Radiation Dose Optimization. **230**(3), 619–628. 10.1148/RADIOL.2303021726 (2004). 10.1148/radiol.2303021726 14739312

[4] Chen, M., Pu, Y. F. & Bai, Y. C. Low-dose CT image denoising using residual convolutional network with fractional TV loss. *Neurocomputing*. **452**, 510–520. 10.1016/J.NEUCOM.2020.10.004 (2021).

[5] Yuan, Q. et al. Edge-preserving median filter and weighted coding with sparse nonlocal regularization for low-dose CT image denoising algorithm. *J. Healthc. Eng.* **2021**. 10.1155/2021/6095676 (2021). 10.1155/2021/6095676 PMC8331292 34354808

[6] Chyophel Lepcha, D., Goyal, B. & Dogra, A. Low-dose CT image denoising using sparse 3d transformation with probabilistic non-local means for clinical applications. **2023**. 10.1080/13682199.2023.2176809 (2023).

[7] Balda, M., Hornegger, J. & Heismann, B. Ray contribution masks for structure adaptive sinogram filtering. *IEEE Trans. Med. Imaging*. **31**, 1228–1239. 10.1109/TMI.2012.2187213 (2012). 22333988 10.1109/TMI.2012.2187213

[8] Chen, G. H., Tang, J. & Leng, S. Prior image constrained compressed sensing (PICCS): A method to accurately reconstruct dynamic CT images from highly undersampled projection data sets. *Med. Phys.* **35**(2), 660–663. 10.1118/1.2836423 (2008). 10.1118/1.2836423 PMC2655145 18383687

[9] Xu, Q. et al. Low-dose X-ray CT reconstruction via dictionary learning. *IEEE Trans. Med. Imaging*. **31** (9), 1682–1697. 10.1109/TMI.2012.2195669 (2012). 22542666 10.1109/TMI.2012.2195669 PMC3777547

[10] Kim, K. et al. Mar., Sparse-view spectral CT reconstruction using spectral patch-based low-rank penalty. *IEEE Trans. Med. Imaging*. **34**(3), 748–760. 10.1109/TMI.2014.2380993 (2015). 10.1109/TMI.2014.2380993 25532170

[11] Niu, S. et al. Sparse-view x-ray CT reconstruction via total generalized variation regularization. *Phys. Med. Biol.* **59** (12), 2997. 10.1088/0031-9155/59/12/2997 (2014). 10.1088/0031-9155/59/12/2997 PMC4219274 24842150

[12] Kim, W., Jeon, S. Y., Byun, G., Yoo, H. & Choi, J. H. A systematic review of deep learning-based denoising for low-dose computed tomography from a perceptual quality perspective. *Biomed. Eng. Lett.* **14** (6), 1153–1173. 10.1007/S13534-024-00419-7/TABLES/2 (2024). 10.1007/s13534-024-00419-7 PMC11502640 39465112

[13] Li, K. et al. Feb., Low-dose CT via convolutional neural network. *Biomed. Opt. Express*. **8**(2), 679–694. 10.1364/BOE.8.000679 (2017). 10.1364/BOE.8.000679 PMC5330597 28270976

[14] Chen, H. et al. Low-dose CT with a residual encoder–decoder convolutional neural network. *IEEE Trans. Med. Imaging*. **36** (12), 2524–2535. 10.1109/TMI.2017.2715284 (2017). 10.1109/TMI.2017.2715284 PMC5727581 28622671

[15] Wu, D., Kim, K., Fakhri, G. E. & Li, Q. A cascaded convolutional neural network for X-ray Low-dose CT image denoising, May 2017, Accessed: Dec. 14, 2024. https://arxiv.org/abs/1705.04267v2.

[16] Wolterink, J. M., Leiner, T., Viergever, M. A. & Išgum, I. Generative adversarial networks for noise reduction in low-dose CT. *IEEE Trans. Med. Imaging*. **36** (12), 2536–2545. 10.1109/TMI.2017.2708987 (2017). 10.1109/TMI.2017.2708987 28574346

[17] Yang, Q. et al. Low-dose CT image denoising using a generative adversarial network with wasserstein distance and perceptual loss. *IEEE Trans. Med. Imaging*. **37**(6), 1348–1357. 10.1109/TMI.2018.2827462 (2018). 10.1109/TMI.2018.2827462 PMC6021013 29870364

[18] Shan, H. et al. 3-D convolutional Encoder-Decoder network for Low-Dose CT via transfer learning from a 2-D trained network. *IEEE Trans. Med. Imaging*. **37** (6), 1522–1534. 10.1109/TMI.2018.2832217 (2018). 10.1109/TMI.2018.2832217 PMC6022756 29870379

[19] Fan, F. et al. Quadratic autoencoder (Q-AE) for low-dose CT denoising. *IEEE Trans. Med. Imaging*. **39** (6), 2035–2050. 10.1109/TMI.2019.2963248 (2020). 10.1109/TMI.2019.2963248 PMC7376975 31902758

[20] Zhao, T., Hoffman, J., McNitt-Gray, M. & Ruan, D. Ultra-low-dose CT image denoising using modified BM3D scheme tailored to data statistics. *Med. Phys.* **46**(1), 190–198. 10.1002/MP.13252 (2019). 10.1002/mp.13252 30351450

[21] Trung, N. T., Hoan, T. D., Trung, N. L. & Luong, M. Low-dose CT image denoising using image decomposition and sparse representation. *REV J. Electron. Commun.* **9**, 3–4. 10.21553/REV-JEC.238 (2020).

[22] Li, S. & Wang, G. Low-dose CT image denoising using parallel-clone networks. 10.48550/arxiv.2005.06724 (2020).

[23] Zhang, J. et al. Task-oriented low-dose CT image denoising. In *Lecture Notes in Computer Science (including subseries Lecture Notes in Artificial Intelligence and Lecture Notes in Bioinformatics)*, vol. 12906 LNCS, 441–450. 10.1007/978-3-030-87231-1_43/COVER (2021).

[24] Bai, T., Wang, B., Nguyen, D. & Jiang, S. Probabilistic self-learning framework for low-dose CT denoising. *Med. Phys.* **48** (5), 2258–2270. 10.1002/MP.14796 (2021). 10.1002/mp.14796 33621348

[25] Trung, N. T., Trinh, D. H., Trung, N. L. & Luong, M. Low-dose CT image denoising using deep convolutional neural networks with extended receptive fields. In *Signal, Image and Video Processing 2022*, 1–9. 10.1007/S11760-022-02157-8 (2022).

[26] Wang, D. et al. CTformer: Convolution-free Token2Token dilated vision transformer for low-dose CT denoising. *Feb*10.48550/arxiv.2202.13517 (2022). 10.1088/1361-6560/acc000 36854190

[27] Khmag, A. & Kamarudin, N. Natural image deblurring using recursive deep convolutional neural network (R-DbCNN) and second-generation wavelets. In *Proceedings of the IEEE International Conference on Signal and Image Processing Applications, ICSIPA 2019*, 285–290. 10.1109/ICSIPA45851.2019.8977756 (2019).

[28] Shukla, P. K. et al. Multiobjective genetic algorithm and convolutional neural network based COVID-19 identification in chest X-ray images. *Math. Probl. Eng.* **2021**. 10.1155/2021/7804540 (2021).

[29] Lilhore, U. K. et al. Hybrid model for detection of cervical Cancer using causal analysis and machine learning techniques. *Comput. Math. Methods Med.* **2022**10.1155/2022/4688327 (2022). 10.1155/2022/4688327 PMC9095387 35572826

[30] Dhiman, P. et al. A novel deep learning model for detection of severity level of the disease in citrus fruits, *Electronics*. **11**, 495. 10.3390/ELECTRONICS11030495 (2022).

[31] Yang, L. et al. Low-dose CT denoising via sinogram inner-structure transformer. *IEEE Trans. Med. Imaging*. 10.1109/TMI.2022.3219856 (2022). 36331637 10.1109/TMI.2022.3219856

[32] Han, Z. et al. A dual-encoder-single-decoder based low-dose CT denoising network, *IEEE J. Biomed. Health Inform.* **26**(7), 3251–3260. 10.1109/JBHI.2022.3155788 (2022). 10.1109/JBHI.2022.3155788 35239495

[33] Kim, W., Lee, J., Kang, M., Kim, J. S. & Choi, J. H. Wavelet subband-specific learning for low-dose computed tomography denoising, *PLoS One*. **17**(9), e0274308. 10.1371/JOURNAL.PONE.0274308 (2022). 10.1371/journal.pone.0274308 PMC9462582 36084002

[34] Li, S. et al. An adaptive self-guided wavelet convolutional neural network with compound loss for low-dose CT denoising. *Biomed. Signal. Process. Control*. **75**, 103543. 10.1016/J.BSPC.2022.103543 (2022).

[35] Li, Z. et al. Multi-Scale feature fusion network for Low-Dose CT denoising. *J. Digit. Imaging*. 1–18. 10.1007/S10278-023-00805-0/METRICS (2023). 10.1007/s10278-023-00805-0 PMC10406773 36914854

[36] Wang, J. et al. Jan., Domain-adaptive denoising network for low-dose CT via noise estimation and transfer learning, *Med. Phys.* 50(1), 74–88. 10.1002/MP.15952 (2023). 10.1002/mp.15952 36018732

[37] Yan, R. et al. Image denoising for low-dose CT via convolutional dictionary learning and neural network. *IEEE Trans. Comput. Imaging*. **9**, 83–93. 10.1109/TCI.2023.3241546 (2023).

[38] Isola, P., Zhu, J. Y., Zhou, T. & Efros, A. A. Image-to-image translation with conditional adversarial networks. In *Proceedings—30th IEEE Conference on Computer Vision and Pattern Recognition, CVPR*, vol. 2017-January, 5967–5976. 10.48550/arxiv.1611.07004 (2017).

[39] Yi, X. & Babyn, P. Sharpness-aware low-dose CT denoising using conditional generative adversarial network. *J. Digit. Imaging*. **31**(5), 655–669. 10.1007/S10278-018-0056-0 (2018). 10.1007/s10278-018-0056-0 PMC6148809 29464432

[40] Ma, Y. et al. Low-dose CT image denoising using a generative adversarial network with a hybrid loss function for noise learning. *IEEE Access.* **8**, 67519–67529. 10.1109/ACCESS.2020.2986388 (2020).

[41] Kwon, T. & Ye, J. C. Cycle-free cyclegan using invertible generator for unsupervised low-dose CT denoising. *IEEE Trans. Comput. Imaging*. **7**, 1354–1368. 10.1109/TCI.2021.3129369 (2021).

[42] Du, W., Chen, H., Yang, H. & Zhang, Y. Disentangled generative adversarial network for low-dose CT. *EURASIP J. Adv. Signal Process*. **1**, 1–16. 10.1186/S13634-021-00749-Z/TABLES/4 (2021).

[43] Huang, Z., Zhang, J., Zhang, Y. & Shan, H. Generative adversarial networks with dual-domain U-Net-based discriminators for low-dose CT denoising. *IEEE Trans. Instrum. Meas.* **71**10.1109/TIM.2021.3128703 (2022).

[44] Lepcha, D. C. et al. A constructive non-local means algorithm for low-dose computed tomography denoising with morphological residual processing. *PLoS One*. **18** (9), e0291911. 10.1371/JOURNAL.PONE.0291911 (2023). 10.1371/journal.pone.0291911 PMC10529561 37756296

[45] Han, Y. et al. LDCT image denoising algorithm based on two-dimensional variational mode decomposition and dictionary learning, *Sci. Rep. ***14**(1), 1–17. 10.1038/s41598-024-68668-1 (2024). 10.1038/s41598-024-68668-1 PMC11289268 39080367

[46] Wang, L. et al. A dual encoder LDCT image denoising model based on cross-scale skip connections. *Neurocomputing*. **613**, 128741. 10.1016/J.NEUCOM.2024.128741 (2025).

[47] Li, Q. et al. Feb., Unpaired low-dose computed tomography image denoising using a progressive cyclical convolutional neural network. *Med. Phys*. **51**(2), 1289–1312. 10.1002/MP.16331 (2024). 10.1002/mp.16331 36841936

[48] Wang, Y. et al. Scale-sensitive generative adversarial network for low-dose CT image denoising. *IEEE Access.* **12**, 98693–98706. 10.1109/ACCESS.2024.3425606 (2024).

[49] Buades, A., Coll, B. & Morel, J. M. A non-local algorithm for image denoising. In *Proceedings—2005 IEEE Computer Society Conference on Computer Vision and Pattern Recognition, CVPR 2005*, vol. II, 60–65. 10.1109/CVPR.2005.38 (2005).

[50] Dabov, K., Foi, A., Katkovnik, V. & Egiazarian, K. Image denoising by sparse 3-D transform-domain collaborative filtering. *IEEE Trans. Image Process.* **16**(8), 2080–2095. 10.1109/TIP.2007.901238 (2007). 10.1109/tip.2007.901238 17688213

[51] Tian, C. et al. Deep learning on image denoising: an overview. *Neural Netw.* **131**, 251–275. 10.1016/J.NEUNET.2020.07.025 (2020). 10.1016/j.neunet.2020.07.025 32829002

[52] Khmag, A., Al Haddad, S. A. R., Ramlee, R. A., Kamarudin, N. & Malallah, F. L. Natural image noise removal using nonlocal means and hidden Markov models in transform domain. *Visual Comput.* **34** (12), 1661–1675. 10.1007/S00371-017-1439-9/TABLES/4 (2018).

[53] Khmag, A. Additive Gaussian noise removal based on generative adversarial network model and semi-soft thresholding approach. *Multimed. Tools Appl.* **82** (5), 7757–7777. 10.1007/S11042-022-13569-6/TABLES/4 (2023).

[54] Buades, A., Coll, B. & Morel, J. M. Jul., A Review of image denoising algorithms, with a new one. **4**(2), 490–530. 10.1137/040616024 (2006).

[55] Gilboa, G. & Osher, S. Nov., Nonlocal operators with applications to image processing. **7**(3), 1005–1028. 10.1137/070698592 (2008).

[56] Hou, Y. et al. NLH: A blind pixel-level non-local method for real-world image denoising. *IEEE Trans. Image Process.* **29**, 5121–5135. 10.1109/TIP.2020.2980116 (2020).

[57] Nam, S., Hwang, Y., Matsushita, Y. & Kim, S. J. A holistic approach to cross-channel image noise modeling and its application to image denoising. In *Proceedings of the IEEE Computer Society Conference on Computer Vision and Pattern Recognition*, vol. 2016-December, 1683–1691. 10.1109/CVPR.2016.186 (2016).

[58] Stankovir, R. S. & Falkowski, B. J. The Haar wavelet transform: its status and achievements. *Comput. Electr. Eng.* **29** (1), 25–44. 10.1016/S0045-7906(01)00011-8 (2003).

[59] Sweldens, W. The lifting scheme: A custom-design construction of biorthogonal wavelets. *Appl. Comput. Harmon Anal.* **3**(2), 186–200. 10.1006/ACHA.1996.0015 (1996).

[60] McCollough, C. TU-FG-207A-04: overview of the low dose CT grand challenge. *Med. Phys.* **43**, 3759–3760. 10.1118/1.4957556 (2016).

[61] Xia, W., Shan, H., Wang, G. & Zhang, Y. Physics-/model-based and data-driven methods for low-dose computed tomography: a survey, *IEEE Signal Process. Mag*. **40**(2), 89–100. 10.1109/MSP.2022.3204407 (2023). 10.1109/msp.2022.3204407 PMC10883591 38404742

[62] Li, Z. et al. Low-dose CT image denoising with improving WGAN and hybrid loss function. *Comput. Math. Methods Med.* **1**, 2973108. 10.1155/2021/2973108 (2021). 10.1155/2021/2973108 PMC8416402 34484414

[63] Lepcha, D. C., Goyal, B., Dogra, A., Sharma, K. P. & Gupta, D. N. A deep journey into image enhancement: A survey of current and emerging trends. *Inform. Fusion*. **93**, 36–76. 10.1016/J.INFFUS.2022.12.012 (2023).

[64] Lepcha, D. C., Goyal, B., Dogra, A. & Goyal, V. Image super-resolution: A comprehensive review, recent trends, challenges and applications. *Inform. Fusion*. **91**, 230–260 (2023).

[65] Wang, Z., Bovik, A. C., Sheikh, H. R. & Simoncelli, E. P. Image quality assessment: From error visibility to structural similarity. *IEEE Trans. Image Process.* **13**(4), 600–612. 10.1109/TIP.2003.819861 (2004). 10.1109/tip.2003.819861 15376593

